# Use of Templates to Identify Source of Norovirus Outbreak

**DOI:** 10.3201/eid1505.081625

**Published:** 2009-05

**Authors:** Juventila Liko, William E. Keene

**Affiliations:** Oregon Public Health Division, Portland, Oregon, USA

**Keywords:** Enteric infections, viruses, norovirus, disease outbreaks, oysters, foodborne, questionnaire templates, investigation methods, letter

**To the Editor**: On November 22, 2006, an infection control nurse notified the Marion County (Oregon) Health Department about acute gastroenteritis among persons who had attended a reception at a medical facility on November 16, 2006. With a holiday weekend only hours away, the county health department asked the state health department to join the outbreak investigation.

After interviewing the caterer, organizers, and several attendees, we modified a questionnaire template to reflect potential exposures. Using this questionnaire, we conducted a retrospective cohort study by telephone among reception attendees identified from a ticket list. We defined a case of acute gastroenteritis as reported vomiting or diarrhea (>3 loose stools within a 24-hour period) within 18–72 hours of the event.

Sanitarians inspected the facility and the caterer’s kitchen. We traced implicated oysters (the source of the outbreak) through distribution records; screened stool specimens for norovirus by RT-PCR; tested oysters from the implicated lot for norovirus by qRT-PCR; entered data into a custom outbreak database template; calculated relative risks (RRs) and 95% confidence intervals (CIs) using Epi Info (www.cdc.gov/epiinfo); and assessed the significance of the association between acute gastroenteritis and consumption of implicated oysters by the χ^2^ or Fisher exact test.

Approximately 200 persons attended the reception. We called all households on the reception ticket list with identifiable phone numbers and reached a convenience sample of 66 attendees from 50 households. We determined that 10 had cases of acute gastroenteritis, 53 had no symptoms, and 3 (who were excluded from the analysis) had minor symptoms. The median incubation period was 36 hours (range 31–63 hours). None of the 10 attendees with acute gastroenteritis sought medical attention; stool specimens from 2 of them tested positive for norovirus (1 positive for genogroup II and 1 positive for both I and II).

Illness was associated with consumption of raw oysters on the half shell (RR 11.8; 95% CI 2.8–50; p = 0.0001), which was reported by 8 of the 10 attendees with acute gastroenteritis. No other foods were associated with illness. No significant breaches in food-handling procedures were identified. The only food handler who reported illness had eaten several oysters at the event and became ill 36 hours later.

The oysters had been individually quick frozen on the half shell and packed loosely in cartons after being harvested in South Korea by growers approved by the US Food and Drug Administration. For the reception, a single 6-kg box of oysters was thawed and served raw. The box was from a shipment of 2,200 boxes legally imported in October 2006. Boxes from the same shipment had been distributed to 5 states. Oysters from 4 other cartons were consumed (some cooked) at 2 other Oregon locations. Public health officials in other states were notified and asked to report any related illnesses; none were identified.

Noroviruses (genogroups I and II) were detected in oysters from an intact carton of the implicated lot. Sequencing was not attempted. The implicated lot was voluntarily recalled by the national distributor; most of the lot was embargoed or recalled before the oysters were consumed.

Oysters are a recurrent source of outbreaks and sporadic cases of norovirus infection, vibriosis, and other infections (*1*) because they are frequently eaten raw or undercooked (*2*). Microbial monitoring of oyster harvest areas reduces but does not eliminate the risk for disease associated with consumption of raw or undercooked oysters (*3*).

Oysters from many parts of the world have been implicated in previous norovirus outbreaks (*3*–*6*), including similar norovirus outbreaks in New Zealand in 2004 and 2006 caused by consumption of frozen oysters from South Korea (*6*). In addition, norovirus was detected in 10% of imported oysters in Hong Kong (*7*), and adenoviruses or enteroviruses were identified in 80% of 1 sample of oysters from South Korea (*8*).

Although widely distributed commercial foods are rarely implicated as a source of norovirus infections, oysters (*3*) and raspberries (*9*) are notable exceptions. Without timely subtyping of virus specimens and a PulseNet-like data-sharing system, cluster linkage is unlikely. Norovirus infections are rarely confirmed by laboratory tests, and sporadic cases are rarely considered notifiable. The outbreak we described was recognized and reported because illnesses clustered in 1 workplace. However, even when outbreaks are reported, they are not always investigated thoroughly. The conventional wisdom that many, if not most, foodborne norovirus outbreaks are caused by contamination at the point of service (*10*) may discourage thorough epidemiologic investigations of these outbreaks.

Because thorough outbreak investigations are time-consuming and gastroenteritis outbreaks are common, resource issues often affect decisions about how intensively to pursue investigations. Our use of integrated questionnaire, data entry, and analysis templates (www.oregon.gov/DHS/ph/acd/keene.shtml) facilitated a quick and efficient response to the outbreak described here. Questionnaire design, interviews, data entry, and analysis were completed within 6 hours of the initial report, and distributors and regulatory agencies worked quickly to recall other oysters from the same source, thus probably preventing additional illnesses. We believe that widespread use of such templates would increase the number of outbreaks that could be investigated thoroughly.
